# Antagonism of CGRP Signaling by Rimegepant at Two Receptors

**DOI:** 10.3389/fphar.2020.01240

**Published:** 2020-08-20

**Authors:** Kylie S. Pan, Andrew Siow, Debbie L. Hay, Christopher S. Walker

**Affiliations:** ^1^ School of Biological Sciences, University of Auckland, Auckland, New Zealand; ^2^ School of Chemical Sciences, University of Auckland, Auckland, New Zealand; ^3^ Centre for Brain Research, University of Auckland, Auckland, New Zealand; ^4^ Maurice Wilkins Centre for Molecular Biodiscovery, University of Auckland, Auckland, New Zealand; ^5^ Department of Pharmacology and Toxicology, University of Otago, Dunedin, New Zealand

**Keywords:** rimegepant, gepant, antagonist, calcitonin gene-related peptide, amylin, migraine, AMY_1_

## Abstract

The “gepants” are a class of calcitonin gene-related peptide (CGRP) receptor antagonist molecules that have been developed for the prevention and treatment of migraine. Rimegepant is reported to act at the CGRP receptor, has good oral bioavailability, and has had positive clinical trial results. However, there is very little data available describing its receptor pharmacology. Importantly, rimegepant activity at the AMY_1_ receptor, a second potent CGRP receptor that is known to be expressed in the trigeminovascular system, has not been reported. The ability of rimegepant to antagonize activation of human CGRP, AMY_1_, and related adrenomedullin receptors was determined in transfected in Cos7 cells. Rimegepant was an effective antagonist at both the CGRP and AMY_1_ receptor. The antagonism of both CGRP and AMY_1_ receptors may have implications for our understanding of the mechanism of action of rimegepant in the treatment of migraine.

## Introduction

Rimegepant (BHV-3000; formerly BMS-927711) is a small molecule drug that has recently been approved as a treatment for migraine (Scott, 2020). It is a member of the “gepant” class of molecules which antagonize the activity of the neuropeptide calcitonin gene-related peptide (CGRP) (Luo et al., 2012; Hargreaves and Olesen, 2019). CGRP is a key player in migraine (Hargreaves and Olesen, 2019). Monoclonal antibodies and orally bioavailable small molecule antagonists, including rimegepant, are approved as migraine therapeutics (Luo et al., 2012; Hargreaves and Olesen, 2019). These therapeutics reduce CGRP signaling by preventing receptor activation. The canonical CGRP receptor comprises the calcitonin receptor-like receptor (CLR) together with receptor activity-modifying protein (RAMP) 1. CLR also complexes with RAMP2 and RAMP3 to comprise adrenomedullin (AM) receptors 1 and 2 respectively. In addition, RAMPs interact with the calcitonin receptor (CTR) to form AMY_1_, AMY_2_, and AMY_3_ receptors, which have high affinity for amylin. The AMY_1_ receptor also has high affinity for CGRP (Hay et al., 2018). The complex nature of this peptide-receptor family means that understanding the pharmacology of CGRP receptor antagonists and the role of each of these ligands and receptors in migraine is important. Rimegepant is frequently reported as a selective CGRP receptor antagonist (Mullin et al., 2020). However, the rationale for this claim is unclear and primary data describing the receptor pharmacology of rimegepant is scarce. The ability of rimegepant to antagonize CGRP activity at the AMY_1_ receptor has not been published (Hay et al., 2018), although a recent report implied that rimegepant can antagonize this receptor (Mullin et al., 2020). Other “gepants” have activity at the AMY_1_ receptor, and therefore it is likely that this is also the case for rimegepant. Activity at the AM receptors is also unclear for rimegepant. Importantly, both the CGRP and AMY_1_ receptors are reportedly expressed at sites relevant to migraine pathophysiology, notably the trigeminal ganglia (Walker et al., 2015). AM receptors may also be present (Walker and Hay, 2013). However the role of these receptors in migraine is not yet known. Results of clinical studies investigating whether AM or amylin can trigger migraine are awaited to help determine the potential role of AM or AMY receptors in migraine (clinicaltrials.gov: NCT03598075, NCT04111484). Additional pharmacological profiling at of rimegepant at defined receptors is needed to allow more accurate understanding of its mechanism of action.

## Material and Methods

### Chemical Analysis of Rimegepant

Rimegepant was purchased from Ak Scientific (Union City, CA). Other reagents were purchased as reagent grade from Scharlau (Barcelona, Spain), Halocarbon (River Edge, New Jersey), ECP limited (Auckland, New Zealand) or Sigma-Aldrich (St. Louis, Missouri) and used without further purification. Analytical reverse phase high-performance liquid chromatography (RP-HPLC) was performed on a Dionex Ultimate 3000 (Thermo Scientific, Waltham, MA) with a C3 analytical column (Agilent Technologies, Santa Clara, CA; 3.5 μm; 3.0 × 150 mm) using a suitably adjusted gradient of 5% B to 95% B, where solvent A was 0.1% TFA/H_2_O and B was 0.1% TFA/acetonitrile at a flow rate of 0.3 ml/min. ESI-MS spectra were acquired using Agilent Technologies (Agilent) 1260 Infinity LC equipped with an Agilent Technologies 6120 Quadrupole mass spectrometer (Agilent).

### Cell Culture and Transfection

Culture and transient transfection of Cos7 cells were performed as previously described (Bailey and Hay, 2006; Walker et al., 2015; Walker et al., 2018). Briefly, Cos7 cells were co-transfected with plasmids containing human CLR (Hemagglutinin tagged) and human RAMP1 (myc tagged) to form the human CGRP receptor, or the human CTR (CT_(a)_; Hemagglutinin tagged) and human RAMP1 to form the AMY_1_ receptor. For AM receptors, human CLR was co-transfected with RAMP2 (FLAG tagged) or RAMP3 (untagged), generating AM_1_ and AM_2_ receptors, respectively.

### cAMP Assay in Transfected Cells

Assays were performed as previously described (Walker et al., 2018). For inhibition (IC_50_) assays, cells were stimulated with 10 nM *α*CGRP with or without 0.1 nM to 10 µM rimegepant for 15 min at 37°C. For antagonist shift assays (pA_2_ or pK_B_), cells were stimulated with 1 pM to 1 µM agonists (human *α*CGRP, human amylin or human AM) with or without rimegepant (as detailed in the figure) for 15 min at 37°C. All agonist peptides were purchased form American Peptide (Sunnyvale, CA) or synthesized as previously described (Bower et al., 2018).

### Data Analysis

All statistical analysis and curve-fitting were performed using GraphPad Prism 7.0 (GraphPad Software Inc. San Diego, CA). Agonist pEC_50_ values for *α*CGRP and amylin were obtained through a three-parameter logistic fit of the data. Antagonism was determined using two different approaches. IC_50_ values for the ability of rimegepant to block signaling by 10 nM *α*CGRP were determined from individual experiments using four-parameter logistic fits. pA_2_ and pK_B_ values were then determined using Global Schild analysis on individual experiments. F-tests indicated that the Schild slope was not significantly different to 1 and the Schild slope was fixed to 1 for all data sets. The data were normalized and combined for presentation purposes. The mean pEC_50,_ pIC_50_, pA_2_, and pK_B_ values were calculated and significant differences determined using two-tailed unpaired t-tests. Statistical significance was defined as p < 0.05. All data points are the mean ± SEM combined from n independent experiments.

## Results

**Figure 1:**
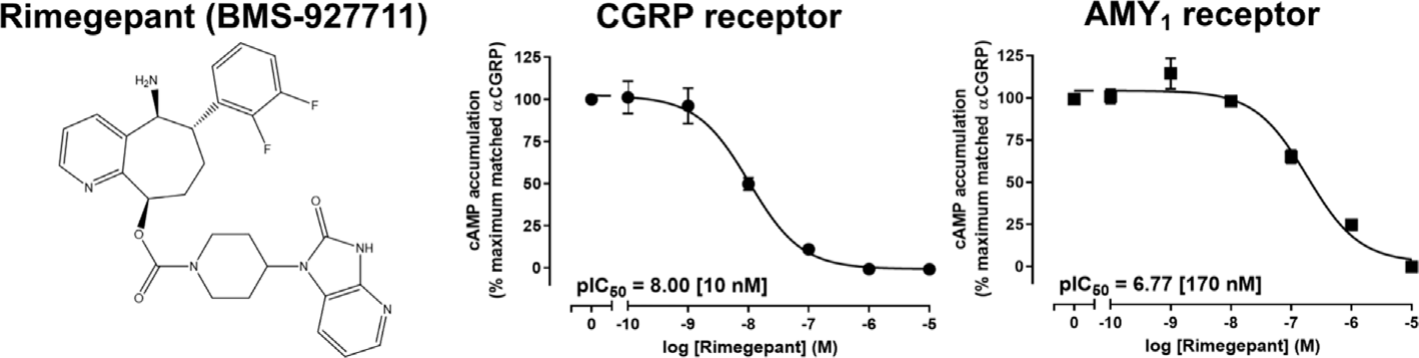
Structure of rimegepant (BMS-927711) and inhibition of human αCGRP-stimulated cAMP accumulation at human CGRP and AMY_1_ receptors by rimegepant in transfected Cos7 cells. Data points are the mean ± SEM of 4 independent experiments.

The purity and molecular weight of rimegepant purchased from Ak Scientific were confirmed using RP-HPLC and ESI-MS (**Supplemental Figures**). The molecular weight was consistent with the reported structure of rimegepant (**Figure 1**) (Luo et al., 2012). Rimegepant antagonism at CGRP and AMY_1_ receptors was compared with two approaches. Initially, its ability to reduce cAMP production in response to 10 nM *α*CGRP was determined. Rimegepant completely blocked activation of the CGRP and AMY_1_ receptors but was approximately 17-fold more effective at blocking the CGRP receptor (p < 0.05; unpaired t-test, t = 18.27, df = 6) (**Figure 1**).

**Figure 2:**
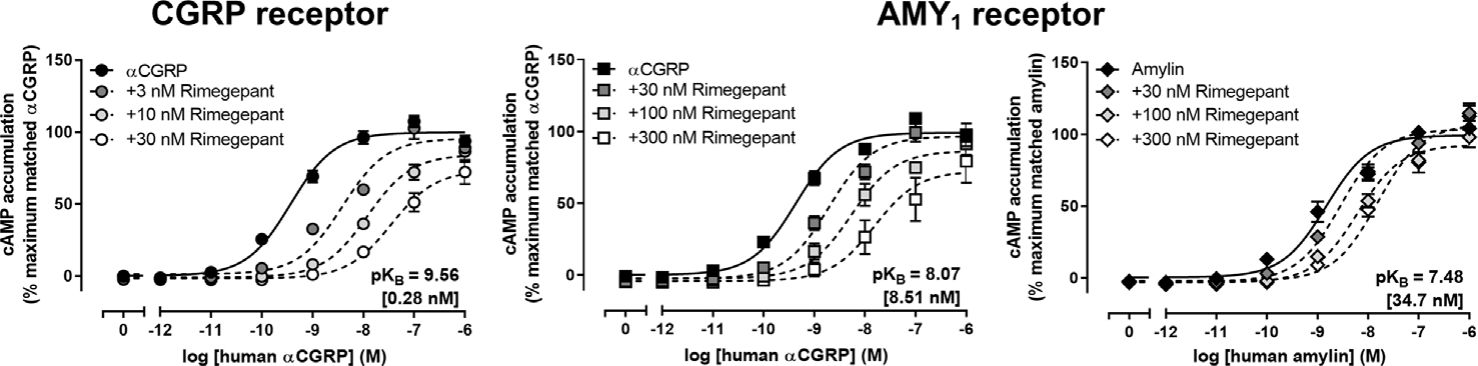
Rimegepant antagonism of human *α*CGRP or human amylin-stimulated cAMP accumulation at human CGRP and AMY_1_ receptors in transfected Cos7 cells. Data are expressed as the percentage of maximum cAMP response. Data points are the mean ± SEM of 4 (*α*CGRP) or five (amylin) independent experiments.

Schild analysis was then conducted with multiple concentrations of rimegepant against a range of *α*CGRP concentrations at both receptors and amylin concentrations at the AMY_1_ receptor (**Figure 2**, **Table 1**). Rimegepant antagonized signaling through both the CGRP and AMY_1_ receptors but was approximately 30-fold more effective at blocking the CGRP receptor (p < 0.05; unpaired t-test, t = 13.32, df = 6). Interestingly, at the AMY_1_ receptor, rimegepant was approximately fourfold less effective at antagonizing amylin signaling than *α*CGRP signaling, with mean pK_B_ values ± s.e.m of 7.48 ± 0.19 (n = 5, amylin) and 8.07 ± 0.11 (n = 4, *α*CGRP), respectively (p < 0.05; unpaired t-test, t = 2.57, df = 7). The ability of rimegepant to block AM signaling at the human AM_1_ and AM_2_ receptors was determined. Rimegepant (10 µM) did not significantly antagonize AM signaling at either the AM_1_ or AM_2_ receptors (**Table 1**).

**Table 1:** Summary data for rimegepant antagonism at human CGRP, AMY_1_, and AM receptors in transfected Cos7 cells.

	Antagonism (pA_2_ or pK_B_)	Fold difference compared to the CGRP receptor
CGRP receptor	9.56 ± 0.02 (4) [0.28 nM]	–
AMY_1_ receptor	8.07 ± 0.11 (4)* [8.51 nM]	30
AM_1_ receptor	<5 (4) [>10 μM]	>36,000
AM_2_ receptor	<5 (4) [>10 μM]	>36,000

The agonist is αCGRP at the CGRP and AMY_1_ receptors and AM at the AM_1_ and AM_2_ receptors. Data points are the mean ± SEM of four independent experiments in parentheses. Corresponding A_2_ and K_B_ values are shown in square brackets. *p < 0.05 by unpaired t-test between CGRP and AMY_1_ receptors. –; no difference.

## Discussion

Rimegepant effectively antagonized *α*CGRP-mediated signaling through the AMY_1_ receptor, in addition to the CGRP receptor. Rimegepant was only 17 or 30-fold more potent at antagonizing *α*CGRP signaling at the CGRP receptor than the AMY_1_ receptor, depending on the experimental approach. This difference is similar to telcagepant and consistent with other gepants, including olcegepant (Hay et al., 2006; Walker et al., 2015; Walker et al., 2018). Gepants share many structural similarities and are known to interact with RAMP1 (Moore and Salvatore, 2012; Hargreaves and Olesen, 2019). Therefore the ability of rimegepant to antagonize two receptors that share this protein is not surprising. In contrast, rimegepant was ineffective at blocking AM activity at the AM_1_ and AM_2_ receptors, which is consistent with other gepants (Moore and Salvatore, 2012).

Interestingly, we observed that rimegepant more potently antagonized *α*CGRP than amylin at the AMY_1_ receptor. Similar reports of agonist-dependent antagonism have been reported for olcegepant, whereby its potency as an antagonist is higher at the AMY_1_ receptor if *α*CGRP is used as the agonist, instead of amylin (Brown et al.; Walker et al., 2018). The reason for this is unclear, but may be related to subtle differences in the ability of gepants to block *α*CGRP and amylin binding or amylin binding to free CTR, which is not blocked by gepants, alongside AMY_1._ These data suggest that the antagonist potency of gepants at the AMY_1_ receptor may be underestimated in several studies. For instance, the potency of ubrogepant and atogepant may be higher at the AMY_1_ receptor than currently reported where only data for amylin is currently available (Hargreaves and Olesen, 2019; Moore et al., 2020).

The potential limitations of this study need to be considered and further studies are required to realize the therapeutic potential of targeting AMY_1_. The current study utilizes receptor component over-expression cell culture models. These models may have exaggerated responses, aberrant or unusual coupling to signaling pathways or in the case of AMY_1_ have potentially higher levels of free CTR than an endogenous cell. The translation of similar findings into primary cultured neuron models suggests that these limitations are minor in this case (Walker et al., 2015; Walker et al., 2018). However, the masking of non-competitive antagonism and similar phenomena by high receptor reserve should be considered, especially when other signaling molecules are being examined (Kenakin et al., 2006; Tasma et al., 2020).

Rimegepant is a clinically effective migraine treatment at a dose of 75 mg (Lipton et al., 2019). This dose is reported to yield ~100 nM free rimegepant in the plasma (Conway et al., 2019). It is therefore reasonable to speculate that the efficacy of rimegepant at alleviating migraine pain could be partially attributed to its action at the AMY_1_ receptor, in addition to the CGRP receptor. The importance of this receptor in migraine is not yet known. Interestingly, eptinezumab, fremanezumab, and galcanezumab (anti-CGRP antibodies) will block *α*CGRP activity at both the CGRP and AMY_1_ receptors (de Vries et al., 2020). To our knowledge, the activity of erenumab (anti-CGRP receptor antibody) has not been examined at a defined AMY_1_ receptor (Shi et al., 2016; Walker and Hay, 2016). The AMY_1_ receptor may already contribute to the activities of these drugs and future efforts should aim to determine its importance in efficacy and side-effect profiles of each class of CGRP blocking agent.

The demonstration that rimegepant is able to effectively antagonize *α*CGRP at the human AMY_1_ receptor serves as evidence that it is necessary to test the gepant class of CGRP receptor antagonists at the AMY_1_ receptor using CGRP as the agonist. These findings highlight the need to consider the AMY_1_ receptor when investigating the efficacy and side-effects of drugs targeting the CGRP system.

## Data Availability Statement

The raw data supporting the conclusions of this article will be made available by the authors, without undue reservation.

## Author Contributions

DH and CW conceived and designed the research. KP and AS performed the experiments. KP and CW analyzed the data. KP, AS, DH, and CW interpreted results of experiments and prepared figures. DH and CW drafted the manuscript. DH and CW edited and revised the manuscript. All authors contributed to the article and approved the submitted version.

## Funding

The authors disclose receipt of the following ﬁnancial support for the research, authorship, and/or publication of this article: This work was supported by a Health Research Fellowship from the Auckland Medical Research Foundation and an Emerging Researcher Start-up Award from the Kelliher Charitable Trust to CW. DH is the recipient of a James Cook Research Fellowship from the Royal Society of New Zealand. CW is the recipient of a Sir Charles Hercus Health Research Fellowship from the Health Research Council, New Zealand. KP was the recipient of a Summer Studentship award from the University of Auckland.

## Conflict of Interest

DH is a consultant for Intarcia, Merck Sharp & Dohme and receives research funding from Living Cell Technologies. CW receives research support from Living Cell Technologies.

The remaining authors declare that the research was conducted in the absence of any commercial or financial relationships that could be construed as a potential conflict of interest.
