# Supplementary material for: Antagonism of CGRP Signaling by Rimegepant at Two Receptors
Source: Front Pharmacol. 2020 Aug 20;11:1240. doi: 10.3389/fphar.2020.01240 (PMC7468408; doi:10.3389/fphar.2020.01240)
Supplement: Supplementary file 1 [file DataSheet_1.pdf]

frontiers

Supplementary Material

Antagonism of CGRP signaling by rimegepant at two receptors

Kylie S Pan[1], Andrew Siow[2], Debbie L Hay[1,3,4,5*], Christopher S Walker[1,3,4*]

[1] School of Biological Sciences, University of Auckland, Auckland, New Zealand.

[2]School of Chemical Sciences, University of Auckland, Auckland, New Zealand.

[3]Centre for Brain Research, University of Auckland, Auckland, New Zealand.

[4] Maurice Wilkins Centre for Molecular Biodiscovery. New Zealand.

[5]Department of Pharmacology and Toxicology, University of Otago, Dunedin, New Zealand.

*** Correspondence:**
Christopher Walker
cs.walker@auckland.ac.nz

Debbie Hay
dl.hay@auckland.ac.nz

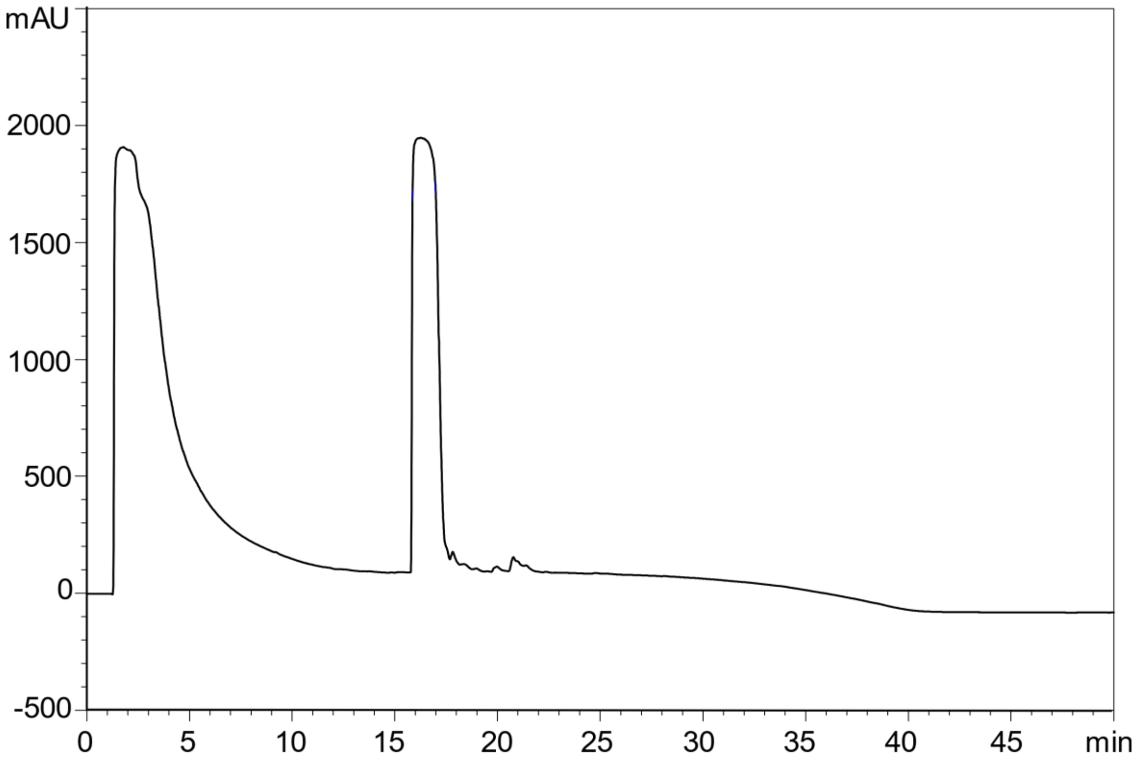

Supplemental Figure 1. Analytical RP-HPLC chromatogram of rimegepant (BMS-927711), t_R = 16.2 min. Chromatographic separations were performed on a Thermo Scientific Dionex Ultimate 3000 HPLC using a Agilent C3, (3.5 µm; 3.0 x 150 mm) at a flow rate of 0.3 mL $min^{-1}$and a linear gradient of 5-95% B in 50 min at room temperature, *ca*. 2% B per min at a flow rate of 0.3 mL/min. Buffer A: H_2O containing 0.1% TFA (*v/v*); Buffer B: acetonitrile containing 0.1% TFA (*v/v*). The peak at 2-5 min corresponds to the absorption of CH_2Cl_2 (solvent).

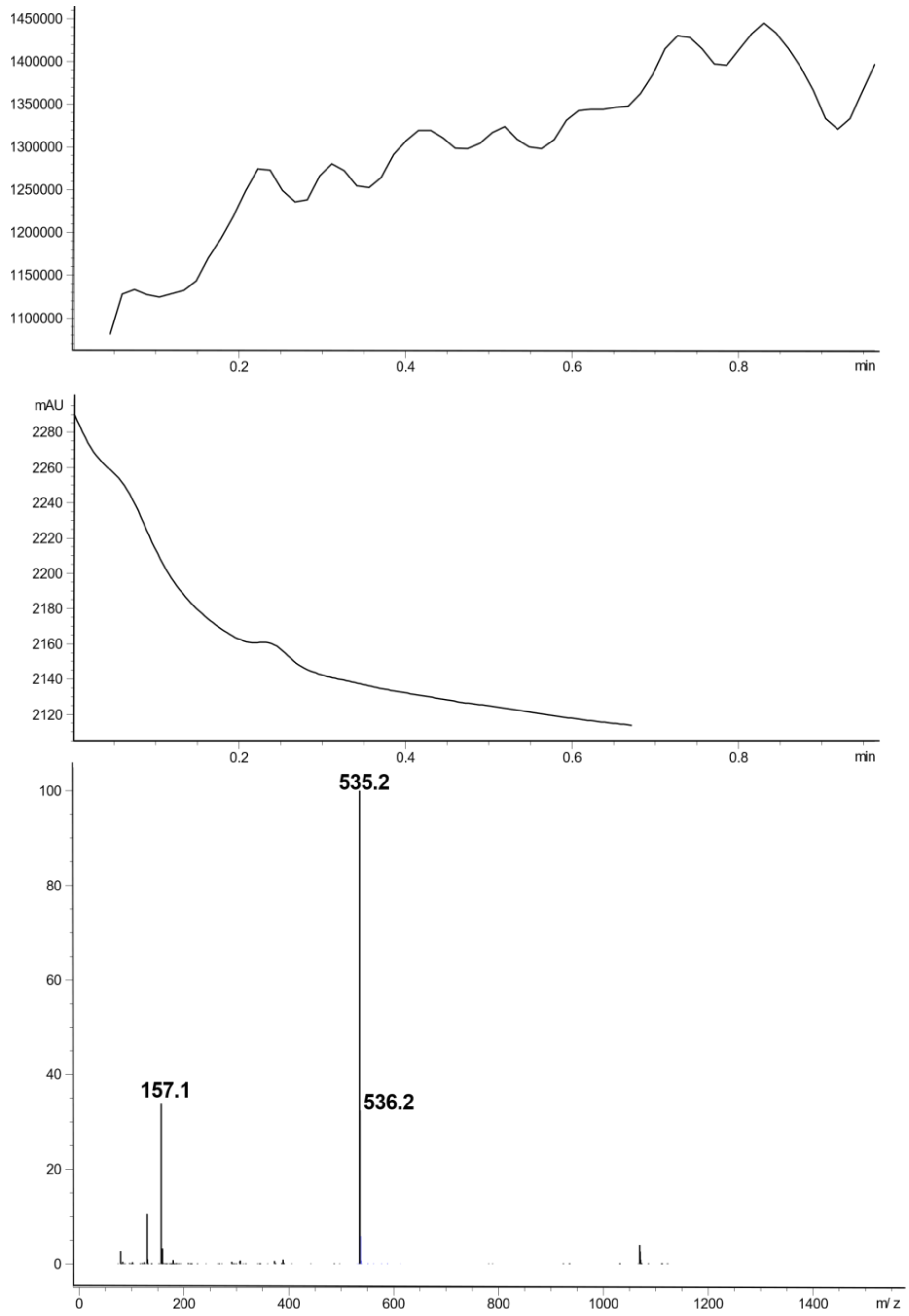

Supplemental Figure 2. ESI-MS profile of rimegepant (BMS-927711); Ion polarity positive, 25 eV. ESI-MS (*m/z* $[M+H]^+$ calculated: 535.5; found: 535.2). The ESI-MS data is consistent with previous reports[1].

1. Luo G, Chen L, Conway CM, Denton R, Keavy D, Signor L, et al. Discovery of (5S,6S,9R)-5-amino-6-(2,3-difluorophenyl)-6,7,8,9-tetrahydro-5H-cyclohepta[b]pyri din-9-yl 4-(2-oxo-2,3-dihydro-1H-imidazo[4,5-b]pyridin-1-yl)piperidine-1-carboxylate (BMS-927711): an oral calcitonin gene-related peptide (CGRP) antagonist in clinical trials for treating migraine. Journal of medicinal chemistry. 2012;55(23):10644-51.
